# Signaling Pathways in Cartilage Repair

**DOI:** 10.3390/ijms15058667

**Published:** 2014-05-15

**Authors:** Erminia Mariani, Lia Pulsatelli, Andrea Facchini

**Affiliations:** 1 Laboratory of Immunorheumatology and Tissue Regeneration/RAMSES, Rizzoli Orthopaedic Institute, via di Barbiano 1/10, Bologna 40136, Italy; E-Mails: lia.pulsatelli@ior.it (L.P.); andrea.facchini@unibo.it (A.F.); 2 Department of Medical and Surgical Sciences, University of Bologna, Bologna 40138, Italy

**Keywords:** cartilage, signaling pathways, TGF-β/BMP, IGF, FGF, HIF, Wnt/β-catenin, NF-κB, MAPK, hedgehog

## Abstract

In adult healthy cartilage, chondrocytes are in a quiescent phase characterized by a fine balance between anabolic and catabolic activities. In ageing, degenerative joint diseases and traumatic injuries of cartilage, a loss of homeostatic conditions and an up-regulation of catabolic pathways occur. Since cartilage differentiation and maintenance of homeostasis are finely tuned by a complex network of signaling molecules and biophysical factors, shedding light on these mechanisms appears to be extremely relevant for both the identification of pathogenic key factors, as specific therapeutic targets, and the development of biological approaches for cartilage regeneration. This review will focus on the main signaling pathways that can activate cellular and molecular processes, regulating the functional behavior of cartilage in both physiological and pathological conditions. These networks may be relevant in the crosstalk among joint compartments and increased knowledge in this field may lead to the development of more effective strategies for inducing cartilage repair.

## 1. Introduction

Articular cartilage is an avascular, highly specialized tissue constituted by an extensive extracellular matrix (ECM) mainly composed of two types of macromolecules, collagen (types II, IX and XI) and proteoglycan (aggrecan) [1]. The biophysical properties of cartilage derive from this highly organized fibrillar framework, that supplies shape, strength, tensile stiffness and compressive resistance to the tissue [2]. The synthesis, maintenance and degradation of ECM proteins are coordinated by chondrocytes, the only resident cell type in cartilage [2].

In healthy adult cartilage, chondrocytes are in a quiescent phase characterized by a fine balance between synthetic and catabolic activities [1]. In ageing, degenerative joint diseases and traumatic injuries of cartilage, a loss of homeostatic conditions and an up-regulation of catabolic pathways occur [1,3].

In osteoarthritis (OA), the most prevalent chronic joint disease, several different factors are recognized as main mediators and/or effectors of progressive cartilage loss, including proinflammatory cytokines (IL-1, TNF) [4–6], chemokines, [7–11], extracellular matrix degrading enzymes, such as metalloproteinases (MMPs) and aggrecanases (A Disintegrin And Metalloproteinase with Thrombospondin Motifs-ADAMTS), which act as downstream key players in the inflammatory signal cascade.

A further hallmark of degenerated cartilage is the modification of chondrocyte differentiation stage. Indeed, chondrocyte phenotype switches toward a hypertrophic phenotype [1,12], thus recapitulating some of the physiological differentiation steps occurring in growth plates and endochondral ossification.

Hypertrophic chondrocytes are characterized by the expression of terminal differentiation markers, including Runt-related transcription factor 2 (RUNX-2), Collagen X, MMP-13 and Indian hedgehog (Ihh) [13–18] and by the acquisition of an “autolytic” phenotype marked by their ability to induce the degradation of pericellular cartilage matrix [13,19]. Conversely, the expression of hypertrophy-related genes is downregulated by cartilage protective mechanism such as Dickkopf (Dkk-1), which acts by inhibiting Wnt signaling [20].

The fate of chondrocytes to remain within cartilage or undergo hypertrophic maturation prior to ossification is also subject to complex regulation by the interplay of the FGF, TGF-β, BMP and Wnt signaling pathways. In particular, whereas FGF signaling accelerates the rate of terminal hypertrophic differentiation, BMPs have been shown to hinder this process. Therefore FGF signals act as antagonists of BMP signaling and negatively regulate Ihh expression [21].

Given the well-known limited ability of cartilage to self-regenerate, damage induced by aging, injury and pathological conditions undergoes unsuccessful reparative processes, resulting in a nonfunctional newly formed tissue [22,23]. Due to this lack of self-regeneration, several strategies have been applied to improve the treatment of cartilage lesions: surgical techniques, transplantation approaches, tissue engineering and bioregeneration technologies [24].

Since cartilage differentiation and maintenance of homeostatic conditions are finely tuned by a complex network of signaling molecules and biophysical factors, shedding light on these mechanisms appears to be extremely relevant for both the identification of key pathogenic factors as specific therapeutic targets and the development of biological approaches for improving cartilage regeneration.

This review focuses on the crucial pathways that drive cartilage physiopathology. In particular, we will discuss the critical signals that can activate cellular and molecular processes in both cartilage regeneration and degradation: transforming growth factor-β (TGF-β), bone morphogenetic proteins (BMPs), fibroblast growth factors (FGFs), hypoxia-related factors (HIF), Wnt/β-catenin, nuclear factor kappa B (NF-κB), mitogen-activated protein kinase (MAPK) and hedgehog (Hh) cascades.

## 2. Transforming Growth Factor-β (TGF-β), Bone Morphogenetic Protein (BMP), Insulin Growth Factor (IGF) and Fibroblast Growth Factor (FGF) Pathways

The development, growth, maintenance and repair of articular cartilage are controlled by several very stringent signaling pathways, triggered by numerous bioactive growth factors. They are needed to properly regulate chondrogenesis and a combination of them, working synergistically, is probably necessary for the cartilage reparative procedure, enhancing of cartilage matrix synthesis as in the case of BMP-7 and IGF-I [25], and differential regulation of their own and each other’s gene expression and protein production *in vitro* as for IGF-I, FGF-2, and TGF-β [26]. Among these molecules those of the TGF-β family play a prominent role (reviewed by [27]).

The TGF-β superfamily is comprised of more than forty members, also including the BMPs [28]. It is noteworthy that TGF-β1 is one of the main molecules considered to be anabolic for cartilage [29–31], together with Insulin Growth Factor (IGF)-1 [32], Fibroblast Growth Factor (FGF)-2 [33] and BMP-7 [34]. Conversely, TGF-β has been shown to be involved in cartilage degeneration during ageing and OA. These conflicting actions depend on the alternative activation of different signaling pathways [13,19,35–37].

TGF-β signals via its type II receptor which then engages the type I receptors. These receptors are called activin-like kinase (ALK)1 and ALK5 [35,38–40] and respectively they activate the Smad 1-5-8 pathway and phosphorylate Smad 2–3 [35,39].

Strong evidence suggests that these two activation pathways are master regulators of chondrocyte phenotypic change and differentiation progression [35]. This hypothesis is based mainly on animal studies but it is corroborated by confirmation studies on human OA tissues [29,30,41,42].

ALK5 activation by TGF-β engagement and subsequent signaling via Smad 2–3 contributes to the maintenance of the stable quiescent phase of chondrocytes and the induction of aggrecan and collagen II production. Smad 2 and 3 exert an inhibitory effect on chondrocyte hypertrophy [30,43], which represents the phenotypic hallmark of terminal differentiated chondrocytes. A similar phenotypic modification occurs in OA and also in ageing chondrocytes [13,44,45] and it has been shown to be associated with a reduced expression of ALK5 leading to a break of the chondrocyte quiescent state and the induction of the terminal differentiation of chondrocytes [13].

Conversely, the activation of the Smad 1-5-8 pathway by ALK1 cooperates with RUNX-2 to stimulate hypertrophic differentiation with the consequent production of Collagen X, MMP13, osteopontin, alkaline phosphatase, osteocalcin and vascular endothelial growth factor (VEGF) by chondrocytes [42,46].

Recent elegant studies by the group at Radboud University have demonstrated a shift in the ALK1/ALK5 ratio occurring in ageing and during OA both in humans and in mice [35]. In ageing and in OA, a loss of the TGF-β receptor ALK5 reduced the phosphorylation of Smad 2–3, whereas only a small decrease in ALK1 expression is documented [35], therefore a relative predominance of Smad 1-5-8 signaling is operating in ageing and OA cartilage, thus promoting the hypertrophic differentiation.

The above-mentioned studies underline the complexity of the various actions of TGF-β in cartilage homeostasis and OA development. A great deal of data from mouse studies can be applied to human pathology with caution. Nonetheless, during OA development (and in ageing) chondrocytes are under the simultaneous influence of various stimuli that probably induce reciprocal opposing effects, the net sum of which determines the final metabolic response.

BMPs are involved in all phases of chondrogenesis and are essential for the endochondral bone formation (reviewed by [47]). These activities are carried out by regulating Smad 1-5-8 and Smad 4 which are critical transcription regulators [46]. Several BMPs (namely BMP-2, -4–6, -11) have been detected in normal and OA cartilage [48]. Although BMPs are recognized as protective factors, being able to play an important role in regeneration of cartilage, they have been shown to also be involved in chondrocyte hypertrophy and matrix degradation. Indeed, BMP-2 promotes chondrocyte proliferation and matrix synthesis [49–52] and controls chondrogenesis through the regulation of the expression and activity of SRY-related high-mobility-group box transcription factor (SOX) 9 [53–55]. The role of BMP-2 in enhancing cartilage repair and counteracting cartilage damage is also underlined by studies in animals. In a mouse model of IL-1-induced cartilage injury, BMP-2 increased the collagen II and aggrecan expression. Moreover, blocking BMP activity resulted in a reduced synthesis of proteoglycan [52] and increased cartilage damage [56]. Conversely, BMP-2 induces hypertrophic differentiation of chondrocytes and may promote cartilage degradation by elevating MMP-13 expression, as observed in OA cartilage [57,58]. The requirement of BMPs for chondrocyte terminal differentiation is highlighted by the evidence that loss of Smad 1 and 5 or inhibition of the Smad 1/5/8 signaling cascade blocks the differentiation of chondrocytes and leads to severe cartilage defects [59,60].

Among the BMPs synthesized by human chondrocytes, BMP-7 has been shown to have both anabolic and anti-catabolic effects on cartilage, being able to induce extracellular matrix synthesis by chondrocytes and counteract the catabolic effect induced on these cells by IL-1, IL-6 and fibronectin fragments [61–65]. Evidence concerning the critical role of cell-matrix interaction in the regulation of BMP signaling has been reported (reviewed by [66,67]). In cartilage, hyaluronan-dependent pericellular matrix is critical for BMP-7 mediated response [68,69] and a very recent study demonstrated that the expression of CD44, the principal chondrocyte hyaluronan receptor, supports the cellular response to lower concentrations of BMP-7 [69].

Recently, differential actions of BMP-2 and BMP-7 on the outcome of chondrocyte differentiation have been reported: BMP-2 stimulated chondrocyte hypertrophy, whereas BMP-7 prevented chondrocyte hypertrophy and maintained chondrogenic potential [58]. BMP-7 is already marketed as an agent able to speed up bone healing after fracture [70]. Overall these results provide evidence that currently, BMPs can be considered key players in cartilage repair and promising candidates for clinical application in the field of cartilage and bone regenerative medicine [71,72].

The role of IGF-I in articular cartilage metabolism has been extensively investigated in both physiological and pathological conditions [73–75]. When added exogenously to monolayers or explant cultures of normal articular cartilage from a variety of species, IGF-I induces a plethora of anabolic effects and decreases catabolic responses [76,77]. In particular, IGF-I has been reported to predominantly stimulate matrix synthesis with a minor effect on mitotic activity in articular chondrocytes [78,79] and to accelerate proliferation and differentiation of cartilage progenitor cells in cultures of neonatal mandibular condyles [80]. Furthermore, in the presence of IGF-I, the chondrocyte phenotype was maintained and cells did not progress to the hypertrophic stage [81].

The need for IGF-I to maintain articular cartilage integrity is supported by an *in vivo* study in rats in which chronic IGF-I deficiency led to the development of articular cartilage lesions [82]. In animal models, it was shown that IGF-I enhanced the repair of extensive cartilage defects and protected the synovial membrane from chronic inflammation [83,84]. The interactions between IGF-1 and TGF-β1 on the proliferation and differentiation of chondrocytes have been reported by several studies [85–87], which also highlighted the ability of TGF-β to increase the number of IGF-I receptors in chondrocytes without changing their affinity [86].

Fukumoto and coworkers showed that IGF-1 in combination with TGF-β1 enhanced total cartilage production compared with IGF-1 alone. In particular, TGF-β has been found to act early to induce chondrogenesis, whereas IGF-I enhanced and maintained proliferation, increasing total cartilage formation [77]. Similarly, MSC chondrogenic differentiation is induced by IGF-I but is enhanced when IGF-I and TGF-β1 are used in combination [88,89].

However, the ability of chondrocytes to respond to IGF-I decreases with age [90–92] and in OA [91,93,94]. Evidence suggests an uncoupling of IGF-I responsiveness in OA, indicating that, in OA cartilage organ cultures, IGF-I is able to robustly stimulate proteoglycan synthesis at saturating doses, but it is unable to modulate proteoglycan catabolism [95]. Despite the diminished ability of IGF-I to decrease catabolism in aged and OA cartilage, a combination of IGF-I and BMP-7 results in greater repair potential by enhancing matrix synthesis than either growth factor alone [25,96].

Fibroblast growth factors (FGFs) constitute another family of relevant growth factors for cartilage development and homeostasis [97]. In particular, two members of this family, FGF-2 and FGF-18, have been implicated in the regulation of cartilage remodeling [97,98]. Conflicting results have been obtained concerning the effect of FGF-2 on chondrocytes. Different studies have shown a regenerative effect on cartilage defect treated with FGF-2, in animal models [99–102]. These findings might result from the strong mitogenic effect exerted on chondrocytes by this growth factor [103], which, on the other hand, appears to be ineffective at inducing a successful regeneration of cartilage extracellular matrix [103]. In agreement with anti-anabolic/catabolic effects of FGF-2, studies performed on human chondrocytes [104–107] showed the ability of FGF-2 to up-regulate MMP-13, ADAMTS-4, -5, inhibit cartilage matrix production, antagonize activity of anabolic factors (e.g., BMP-7) and stimulate proinflammatory cytokines, such as IL-1 and TNF.

In contrast to FGF-2, the anabolic effect of FGF-18 in cartilage has been well established [108–111]. In porcine and human cartilage, FGF-18 has been shown to act as an inducer of cell proliferation and extracellular matrix synthesis [108]. Furthermore, in a rat OA model, intra-articular injections of FGF-18 increased cartilage formation and reduced cartilage degeneration scores [111].

## 3. Hypoxia-Inducible Factor (HIF) Pathway

Healthy articular cartilage is typically an avascular tissue. Chondrocyte survival in this hypoxic condition requires the activation of adaptive strategies that promote and sustain tissue function in a low oxygen environment. Hypoxic response is mainly mediated by hypoxia inducible factor (HIF) family members (HIF-1α, -2α, -3α). The first member of this transcription factor family to be identified was HIF-1α [112].

In the presence of normal oxygen levels, HIF-1α is hydroxilated and degraded, whereas, under hypoxic conditions, hydroxilation is inhibited and HIF-1α is free to form a heterodimer with the constitutive HIF-1β unit. This complex binds specific target gene consensus sequences and promotes their transcription [113]. In cartilage, HIF-1α has been shown to be essential for chondrocyte growth arrest and survival [114] and appears to be involved in the regulation of angiogenetic factor expression, mainly VEGF [114–116], thus modulating an essential step in endochondral bone formation. Furthermore, HIF-1α contributes to the maintenance of ECM homeostasis, inducing the gene expression of two main matrix components: collagen II and aggrecan [117,118].

The crucial role of HIF-1α in controlling the survival of hypoxic cartilage has been highlighted by further experimental evidence, underlining the central importance of HIF-1 in supporting the chondrocyte energy generation, via increased glucose uptake and by regulating the activity of glycolytic enzymes [119–121], the synthesis of cartilage matrix [117] and the activation of a protective mechanism enabling the prevention of chondrocyte cell death induced by IL-1β [121].

In damaged cartilage, HIF-1α expression has been reported in several studies [120–124]. An increasing transcription of HIF-1α in OA cartilage compared to normal samples was shown, particularly in the late-stage of the disease. Consistent with this evidence, subsequent studies reported a growing number of HIF-1α-positive chondrocytes during OA progression [120] and a higher expression of HIF-1α mRNA in degenerated cartilage compared to uninjured cartilage [121].

In addition to hypoxic conditions, HIF-1α expression can be up-regulated by other factors, including inflammatory cytokines (IL-1 and TNF-β), reactive oxygen species and mechanical loading [121,125–131], which are all recognized as key players in cartilage damage.

Since HIF-1α has a pivotal role in supporting chondrocyte survival and cartilage homeostasis, these findings have led researchers to consider HIF-1α as a stress-inducible responder, rather than merely as a hypoxia-inducible factor, which potentially acts in order to maintain the chondroprotective functions challenged by the detrimental conditions occurring in the OA joint environment. Overall, these findings characterize HIF-1α as a key factor for chondrocyte survival promoting compensatory mechanisms in response to catabolic modifications of OA cartilage.

Another member of the hypoxic inducible factor family, HIF-2α, has subsequently been recognized. HIF-2α and HIF-1α have extensive structural homology and both of them are regulated by similar mechanisms [132]. Despite these similarities, these transcriptional factors have been shown to have specific and distinct functions under physiological and pathological conditions [133–135].

In healthy cartilage, hypoxic conditions appear to promote the up-regulation of cartilage matrix genes via HIF-2α-mediated SOX-9 induction [136,137].

The distinct, but complementary roles of HIF-1α and HIF-2α have been reported in a study performed on healthy human cartilage: HIF-2α mediates anabolic responses by directly binding a specific SOX-9 regulatory site, HIF-1α induces anti-catabolic effects by modulating the expression of cartilage degrading enzymes and its inhibitors [138]. Furthermore, HIF-2α has been identified as an extensive regulator of endochondral bone formation [139]. This process is marked by sequential steps, including chondrocyte hypertrophic differentiation (characterized by secretion of collagen X), cartilage degradation (via proteinases, mainly MMP-13) and vascular invasion depending on angiogenic stimuli, such as VEGF [140,141]. HIF-2α appears to be a central regulator of all these steps, directly regulating collagen X, MMP-13 and VEGF expression. In addition, further possible transcription targets of HIF-2α related to endochondral ossification have been identified, namely RUNX-2 and Indian hedgehog proteins [139,142].

Conversely, HIF-2α appears to be highly expressed in degenerated cartilage and strongly implicated in catabolic mechanisms leading to cartilage breakdown and endochondral bone formation, which represents a fundamental pathway leading to osteophyte formation, considered as one of the typical OA outcomes [143]. The role of HIF-2α as catabolic inducer of OA cartilage destruction has been clearly demonstrated in studies by Yang *et al.* [144]. They showed that the adenoviral overexpression of HIF-2α causes progressive cartilage damage directly by up-regulating the expression of various degradative enzymes, including MMP1, MMP3, MMP12, MMP13, ADAMTS4.

The relevance of HIF-2α in OA has been strengthened by additional evidence from human knee joint samples, showing increased expression related to the early and progressive stage of OA development, whereas this factor appears to decrease in the late stage of the disease.

HIFs are also implicated in regulating chondrocyte autophagy. Autophagy is a primary process that degrades and removes damaged and dysfunctional intracellular organelles and molecules, thus protecting cells during stress responses [145–147]. Disorders of this mechanism have been found to contribute to the development of aging-related diseases [148,149], indeed this process appears to be compromised in OA cartilage. The role of HIF-1α and HIF-2α in controlling chondrocyte autophagy has been shown by studies demonstrating the ability of HIF-2α to counterbalance the ability of HIF-1α to accelerate chondrocyte autophagy functions [150].

Even if the conflicting role of HIF-2 needs to be clarified, the balance between HIF-1α/HIF-2α activities clearly contributes to the control of cartilage homeostasis.

## 4. Wnt/β-Catenin Pathway

Wnts (Wingless-type) constitute a large family of 19 secreted glycoproteins involved in the development, growth and homeostasis of different tissues and organs, including joints, bone and cartilage (reviewed in [151–154]). Wnts bind receptors of the Frizzled (FZD) family receptors on the plasma membrane to initiate several distinct cascades classified as either canonical or non-canonical, depending on whether β-catenin is involved.

The Wnt pathway, often referred to as the canonical pathway, is the best described and signals through the β-catenin nuclear effector. It is activated upon the binding of Wnt to Frizzled (FZD) receptors and low density lipoprotein receptor-related protein (LRP) 5/6 co-receptors at the cell surface [155,156]. In the absence of appropriate Wnt ligands, β-catenin is phosphorylated using glycogen synthase kinase 3 (GSK3) and, subsequently, targeted for ubiquitination and degradation via the proteasome [157–159]. However, in the presence of a Wnt ligand, its binding to the receptor complex initiates the activation of an intracellular protein termed Dishevelled (Dvl) which in turn inhibits GSK3 activity [160]. Therefore, β-catenin is not targeted for degradation; it accumulates and translocates to the nucleus and modulates the transcription of target genes.

The Wnt/β-catenin pathway is tightly regulated by several natural extracellular antagonists of Wnt signaling, namely secreted frizzled related proteins (sFRPs), Wnt inhibitory factors (Wifs), Dickkopf (Dkk) factors and sclerostin (SCL). The inhibition of the signaling cascade essentially occurs through two different mechanisms: the direct binding of sFRPs and Wifs to Wnts, that interferes with the FZD receptor interactions and blocking of the canonical pathway performed through Dkk and SCL, binding to the LRP5/6 Wnt coreceptor [158,159].

Chondrogenesis is highly dependent upon cell–ECM and cell–cell adhesion interactions and it has been shown that Wnt signaling is necessary either for the maintenance of mature articular cartilage phenotype, which is characterized by extended cell survival and absence of differentiation towards hypertrophy (schematically reported in Table 1) or for the hypertrophic maturation in the process of endochondral ossification (schematically reported in Table 2) (reviewed by [151,152,154]).

Wnt signaling has recently been shown to play an additional role in crosstalk between cartilage and subchondral bone. During late embryonic and postnatal development, the rate of trabecular bone formation is strongly dependent on the levels of β-catenin expressed by chondrocytes in the lower hypertrophic zone. Wnts have been extensively recognized as key regulators of bone cartilage and joint development and homeostasis [158,161–164].

β-catenin in hypertrophic chondrocytes is necessary for regulating the expression of RANKL to control osteoclast activity in subchondral growth plates. Thus, it may be expected that, during the progression of OA, the observed altered activity of Wnt signaling in chondrocytes may affect the osteoclastic activity in subchondral bone growth plates leading to sclerosis or osteophyte formation at the edges of joints. The alteration of Wnt signaling pathway in chondrocytes appears to modulate key regulatory factors for remodeling of subchondral bone, resulting in its aberrant behavior as observed in the case of OA. Similarly, altered Wnt signaling in subchondral bone may modulate chondrogenic factors that are necessary for maintaining cartilage homeostasis [162].

This evidence has prompted researchers to investigate the potential association between Wnt modifications and OA. Enhanced activation of canonical Wnt pathways in OA human cartilage and in injured cartilage [165–167] has been reported. These findings have been confirmed by further studies performed on animal models that report a relationship between β-catenin signaling activation and OA-like phenotype [168–170].

Genetic studies have identified some components of the Wnt cascade as candidate genes associated with OA. A single nucleotide polymorphism in the sFRP3 gene, a Wnt antagonist, appears to be linked to an increased risk for hip OA [171], whereas an association between a single *LRP5* gene polymorphism and spine OA has been reported [172].

The relevance of Dkk-1 as a key regulator of bone remodeling has been shown. In fact, the blockage of Dkk-1, abrogating Dkk-1-mediated Wnt suppression, reverts from the bone-destructive pattern in a mouse model of RA and induces bone formation and osteophyte growth, thus resembling the bone-forming pattern of OA [173].

Wnt signaling antagonists are also considered as potential biomarkers of OA progression. Elevated Dkk-1 serum levels have been shown to be associated with reduced progression of hip OA in a Caucasian population [174]. Similarly, in patients with knee OA, an inverse correlation between Dkk-1 levels in plasma and synovial fluid and the radiographic severity has been reported [175]. In addition, high serum concentrations of sFRP-3 showed a tendency to be associated with a lower risk of developing hip OA [174].

Extensive studies on Wnt signaling related to bone and cartilage physiology and pathology have undoubtedly led to a body of evidence consistent with a critical role in OA pathogenesis. Nevertheless, considering the complexity of the downstream effects of Wnt and its multiple interplays with several other pathways, further research is needed to better characterize the specific role and relevance of single Wnt agents/antagonists in bone, cartilage and the osteochondral junction.

## 5. Nuclear Factor-Kappa B (NF-κB) Pathway

The NF-κB transcription factors are a family of ubiquitously expressed molecules that regulate a wide range of immune responses, cellular growth, differentiation and survival both in normal and neoplastic conditions (Reviewed by [176–180]). Concerning cartilage, activated NF-κB regulates the expression of several matrix degrading enzymes, thus influencing the amount and remodeling of ECM proteins, and shows indirect positive effects on downstream regulators of terminal chondrocyte differentiation (including β-catenin and RUNX-2).

The transcriptional control exerted by NF-κB, is performed by means of five proteins combined in homo or hetero-dimers (RelA/p65, RelB, c-Rel, NF-κB1/p50 (or p105 as a precursor inactive form of p50) and NF-κB2/p52 (or p100 as a precursor inactive form of p52), whose shared *N*-terminal domain with Rel-homology is crucial for different activities, such as the interaction with specific inhibitory proteins, the dimerization in active complexes and the translocation to nucleus [177,181].

The inactive form of the NF-κB dimers is located in the cell cytoplasm bound to specific inhibitory (I-κB) molecules and translocates into the nucleus following cell activation. The stimulation induces the phosphorylation of I-κB molecules mediated by I-κB kinases (IKKB) that, following ubiquitination, are degraded by a proteasome complex [177].

Two distinct pathways regulate the activation of the NF-κB signaling cascades: the canonical/classical way (mediated by TNF, Toll-like and T-cell receptors) and the alternative/non-canonical one (mediated by B-cell activating factor-BAFF, CD40 and lymphotoxin-b receptors) [180,182–184]. The canonical pathway is crucial for the activation of the innate and inflammatory responses as well as for the inhibition of apoptosis, whereas the alternative pathway is involved in lymphoid organogenesis, B-cell activation and humoral immunity. Once in the nucleus, released NF-κB dimers bind to the promoter region of various genes, including immunomodulatory molecules, cytokines, cyclooxygenase-2 (COX-2), inducible NO-synthase (iNOS) and proteases (MMP), thereby activating their transcription [182,185,186].

The activation of NF-κB signaling in OA chondrocytes is induced by fibronectin fragments, pro-inflammatory mediators (TNF-α or IL-1β), mechanical stress and ageing factors through cytokine and Toll-like membrane receptors [187]. The NF-κB pathway alone or cooperating with other pathways (such as Wnt or BMPs) induces the secretion of various degradative enzymes including various matrix metallo-proteinases [177] and ADAMTS4 and ADAMTS5 [181], leading to articular cartilage destruction [1].

MMP in OA chondrocytes is also induced by various catabolic soluble factors such as IL-1beta, IL-6, IL-8, TNF-α and receptor activator of NF-κB (RANK) ligand (RANKL). These cytokines and chemokines decrease also the synthesis of proteoglycan and collagen and contribute to amplifying NF-κB activation. [6]. NF-κB molecules contribute to the articular changes by inducing type X collagen (involved in chondrocyte hypertrophy) and prostaglandin E2 (PGE2), nitric oxide (NO), nitric oxide synthase (NOS) and cyclooxygenase 2 (COX2), which promote the synthesis of catabolic factors, cartilage inflammation and apoptosis of OA chondrocytes [177,188–190].

The protracted activation of NF-κB influences other regulatory transcription factors, such as HIF-2α, RUNX-2 and E74-like factor 3 (ELF3) [191,192]. ELF3 acts as a central mediator of the inflammatory responses triggered by IL-1, TNFα and LPS and modulates the IL-6 expression induced by LPS *in vivo* [193]. In chondrocytes, ELF3 accounts for the IL-1-mediated repression of the type II collagen [194] and participates in the IL-1-induced MMP-13 expression [195].

The above transcription factors stimulate the production of MMP13 and ADAMTS5 thus favoring the acquisition of chondrocyte hypertrophic phenotype (characterized by chondrocyte calcification and osteophyte formation) [191]. In these conditions, the NF-κB proteins induce the synthesis of BMP2, IL-8, GRO-α and growth arrest and DNA damage-inducible protein 45beta (GADD45beta) effector molecules [196,197] and, together with ELF3 and HIF-2α pathways, induce the expression of type X collagen, MMP9, MMP13, alkaline phosphatase, VEGF and osteocalcin [198].

Besides its role in cartilage destruction, NF-κB regulates many genes including cytokines, chemokines and adhesion molecules that participate in the pathophysiology of synovial inflammation (synovitis) and bone degradation in OA joints [187].

In synovial cells the activation of NF-κB [199] mediated by different stimuli (cytokines, cartilaginous matrix fragments, stress) [200,201] induces the synthesis of various cytokines and chemokines (IL-1β, IL-6, TNF-α, RANKL, IL-8, RANTES), angiogenic factors (VEGF, FGF-b) and degradative enzymes (MMPs1-13, ADAMTS4, ADAMTS5) that lead to further cartilage destruction and increased synovitis [202].

Concerning bone, IL-1, IL-6, and TNF (produced by both chondrocyte and synovial cells) induce RANKL synthesis and the binding between the RANKL osteoclastogenic cytokine and RANK surface receptor activates NF-κB [203] to produce IL-1, IL-6, and PGE2 and then bone resorption [204].

In normal conditions RANK/RANKL interaction is regulated by the OPG decoy receptor that by blocking RANKL promotes bone formation [205], whereas in OA a decreased OPG/RANKL ratio favors osteoclast activity and the resulting bone resorption [206–209].

## 6. Mitogen-Activated Protein Kinase (MAPK) Pathway

The MAPK pathway is known to control the conversion of a vast number of extracellular stimuli into specific cellular responses, such as embryogenesis, cell proliferation, differentiation, and survival (reviewed by [153,210–213]).

Three major classes of MAPKs have been described in mammals: the extracellular signal-regulated kinases (ERK-1/2), the c-jun *N*-terminal kinase (JNK) and p38 [214]. ERKs are activated by mitogens and growth factors, whereas JNK and p38 are activated by inflammatory cytokines (IL-1 and TNF-α), cellular stress (heat and osmotic), reactive oxygen species, and ultraviolet irradiation [215–217].

Activation of ERK, JNK and p38 MAPK signaling cascades coordinates phosphorylation events that result in the activation of transcription factors such as AP-1 (cFos/cJun), ETS, RUNX-2, HIF-2α, and C/EBPβ, which together with NF-κB, regulate the expression of genes involved in catabolic and inflammatory events [218–221].

Several studies have shown the requirement of MAPK signaling pathways, during various phases of cartilage formation and maturation and endochondral ossification [210,222,223].

These pathways have been found to play a distinct role in cartilage matrix synthesis and homeostasis and alterations in these signaling pathways (p38 and ERK-1/2, in particular) are reported to play a prominent role in chondrocyte dysfunction as a part of OA pathogenesis and disease progression [213,224]. The activities of p38 are primarily governed by extensive cross-talk with ERK-1/2, resulting in a reciprocal bidirectional equilibrium between ERK-1/2 and p38 phosphorylation [213].

During normal chondrogenesis, the process of cartilage nodule formation is activated by p38s and repressed by ERK-1/2 [225]. In agreement, a strong activation of the ERK-1/2 pathway was observed in the hypertrophic zone of the growth plate, whereas the inhibition of ERK-1/2 delayed hypertrophic differentiation in growth plate chondrocytes during endochondral ossification [210,213]. Furthermore such cross-talk (activation of ERK-1/2 and deactivation of p38) appears to play a role in the induction of hypertrophic changes observed in normal articular chondrocytes *in vitro*, when co-cultured with subchondral osteoblasts form OA patients or exposed to their conditioned medium [213].

Early and late differentiation processes of osteoblasts are promoted by both p38 and ERK-1/2, whereas all three kinases appear to promote osteoclast differentiation by regulating activator protein 1 (AP-1) as a critical mediator of osteoclastogenesis [223]. Furthermore, ERK1/2 is possibly a central regulator to the effects of pro-inflammatory cytokines IL-1β, IL-6, IL-12, IL-23, and TNF-α that cause joint destruction [226,227].

*In vitro*, IL-1 leads to the activation of ERK, p38 and JNK and is associated with the up-regulated expression of MMPs in chondrocytes and conversely, ERK1/2 inhibition decreases the release of MMP3 and MMP13 by human chondrocytes stimulated with IL-1β [228]. In addition, IL-1β exhibits inhibitory effects on collagen type II and aggrecan whose synthesis is necessary for cartilage repair. Thus, IL-1β disrupts the balance between catabolic and anabolic activities, which may result in articular cartilage destruction [228], MMP13 is capable of cleaving type II collagen, whereas MMP3 is active against other components of the extracellular matrix, such as fibronectin and laminin.

The MAPK/ERK pathway is also involved in the IL-1β-induced decrease in type II collagen and aggrecan expression in engineered cartilage, thus compromising the effective host tissue integration [229]. The altered expression of MMP3, MMP13, type II collagen and aggrecan induced by IL-1β stimulation in human chondrocytes was significantly and similarly reverted by ERK1 or ERK2 knockdown; no isoform was significantly upregulated after the single knockdown of the other kinase, and the combined knockdown displayed synergistic effects [228].

## 7. Hedgehog (Hh)/Smoothened (Smo) Pathway

Another signaling pathway strongly involved in both chondrogenesis and chondrocyte proliferation and differentiation in the growth plate during development [230–235] is the Hedgehog (Hh) pathway. The ligands of this pathway (Indian Hh (Ihh) and Sonic Hh (Shh)) are expressed by chondrocytes in response to mechanical stress (Ihh is a mechanoresponsive gene) [236]. These ligands engage Patched 1 receptor (Ptch1), terminating, in this way, the inhibitory action of Ptch1 on another cell surface receptor called Smoothened (Smo) [237]. The Hh ligand binding to Ptch1 causes Smo localization and accumulation in the chondrocyte cilia [238] and induces the activation of downstream transcription factors called Gli proteins [239], the final result being the expression of RUNX-2 (a master regulator of chondrocyte hypertrophic differentiation) and, indirectly, the expression of the aggrecanase ADAMTS5 [240]. In chondrocytes, Ihh signaling induces the expression of parathyroid hormone-related peptide (PTHrP) ligand [234] and the engagement of the corresponding parathyroid hormone 1 receptor (PTH1R) results in the repression of both Ihh expression and chondrocyte hypertrophy. Thus, the Ihh/PTHrP feedback loop controls the proliferative zone of the growth plate and regulates the rate of hypertrophy [234,235,241].

Similarly to the growth plate, in mature cartilage the Ihh–PTHrP axis regulates the differentiation program of chondrocytes, by coordinating chondrocyte maturation and hypertrophy [242] and participating in maintenance of the physiological functionality of articular cartilage.

Since OA chondrocytes typically recapitulate some differentiation steps occurring in growth plates, the involvement of hedgehog signaling has been addressed by several studies.

Hh signaling appears to be aberrantly activated in OA cartilage, thus promoting the hypertrophic phenotype of chondrocytes and up-regulation of MMP-13, one of the extracellular matrix-degrading enzymes that play a key role as mediators of cartilage damage in OA [243]. The inhibition of Hh signaling reduces disease severity in OA animal models, thus identifying a potential therapeutic target for OA treatment [240].

The Ihh/PTHrP axis modulation has also been explored as a molecular system for promoting cartilage repair. Ihh gene transfer induces chondrogenic differentiation of mesenchymal stem cells (MSC) [244] and the ability of Shh to induce redifferentiation of dedifferentiated chondrocytes by the stimulation of SOX-9, BMP-2, and IGF expression has been recently reported [245].

Furthermore, the presence of PTHrP in cultured MSC is able to suppress the hypertrophy during “*in vitro*” chondrogenesis [246]. These findings underline the potential relevance of this pathway to improve outcomes of cartilage regenerative approaches and tissue engineering therapies.

## 8. Signaling Cross-Talk and Conclusions

The balance between anabolic and catabolic activities in cartilage appears to be driven by many pathways. Over the years, each of these pathways had been extensively investigated and partly clarified, but little is still understood about their interplay.

Nevertheless, it is becoming clear that there is significant cross-talk between the different pathways and that the overall effects on chondrocyte function depend on the balance in activity of multiple signaling proteins.

**Figure 1.**
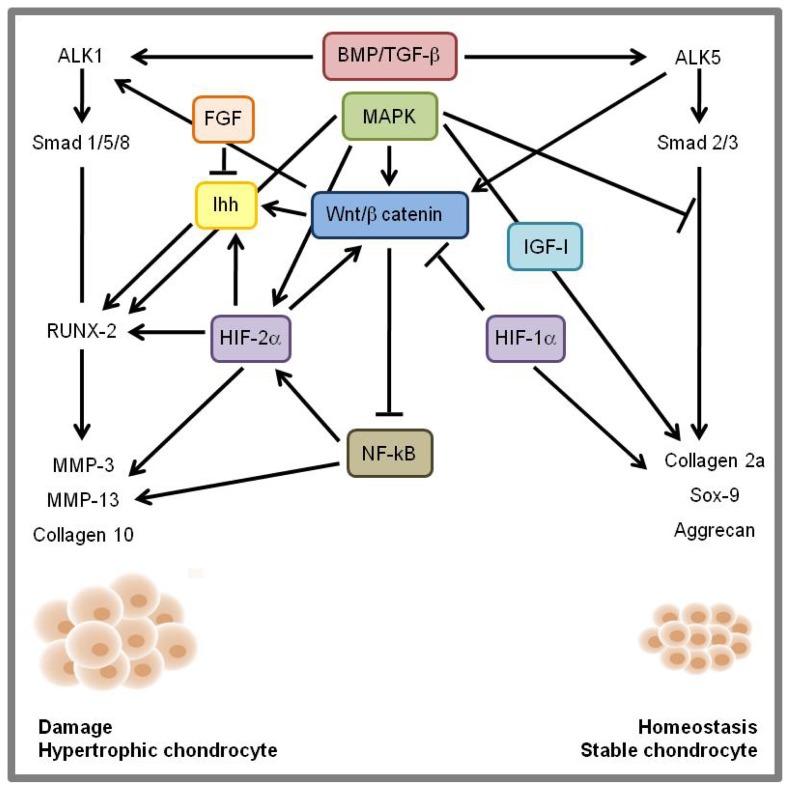
Schematic overview of signaling cross-talk among transforming growth factor-β (TGF-β), bone morphogenetic proteins (BMPs), hypoxia-related factors (HIF), Wnt/β-catenin, nuclear factor kappa B (NF-κB), mitogen-activated protein kinase (MAPK) and Indian hedgehog (Ihh) pathways.

The expression of Wnt/β-catenin negatively regulates NF-κB; on the contrary, canonical Wnt signaling drives TGF-β/BMP—signaling towards the Alk1, Smad 1-5-8 route. Wnt/β-catenin also interacts with Ihh signaling and all these pathways converge on the activation of RUNX-2 and downstream target gene expression (MMP13, MMP3, and Collagen X), thus inducing the hypertrophic chondrocyte phenotype (Figure 1).

In its turn, Wnt/β-catenin is negatively modulated by HIF-1α and positively modulated by MAPK and HIF-2α and by TGF-β/BMP—signaling via the ALK5, Smad 2–3 pathway and, depending on the prevailing cross regulation of the different routes, a good balance (homeostasis/stable chondrocyte) or a deleterious scenario (damage/chondrocyte hyperthropy) may result (Figure 1) [247–250].

The substantial progress made in identifying the pathways regulating anabolic/catabolic response of chondrocytes in developing and adult cartilage has unraveled an ever increasing complexity of the molecular networks involved. The continuous progression of our knowledge regarding the molecular pathways controlling the functional behavior of cartilage, in both physiological and pathological conditions, may lead to the development of more effective strategies for inducing cartilage repair and for the treatment of cartilage-related diseases.

## Figures and Tables

**Table 1.** Role of Wnt signaling during early stages of cartilage development.

**Promoting Effects**	
Wnt5a	Expressed in perichondrium surrounding condensations Recruits mesenchymal stem cells Delays chondrocyte differentiation
Wnt5b	Expressed in pre-hypertrophic chondrocytes and in the perichondrium Promotes initial steps of chondrogenesis in micromass cultures by stimulating cartilage nodule formation Delays terminal differentiation *in vivo*
β-catenin low level	Expressed in chondrogenic mesenchymal condensations Induces expression of Sox9 and promotes chondrogenic differentiation
Dkk1sFRP1	Promotes early chondrogenesis in human mesenchymal stem cells
Wif-1	Expressed in mesenchyme surrounding cartilage elements and articular cartilage Promotes chondrogenic differentiation by neutralizing Wnt3a mediated inhibition of chondrogenesis
**Inhibitory Effects**	
Wnt1	Inhibits cell condensation and thus cartilage formation
Wnt3a	Expressed in early stages of chondrogenesis, decreased when chondrogenic differentiation proceeds Increases self-renewal and decreases apoptosis of MSCs Stabilizes cell-cell adhesion through promoting sustained expression of *N*-cadherin and β-catenin Blocks collagen II expression and proteoglycan deposition
Wnt4	Expressed in developing joint interzone Inhibits cell condensation and thus chondrogenesis condensation
Wnt6	Inhibits chondrogenesis at an early stage prior to chondrogenic differentiation (up-stream of SOX-9)
Wnt7a	Expressed in dorsal ectoderm in developing limb Inhibits chondrogenic differentiation *in vitro* and *in vivo* mediated by MAPK and AP-1 signaling Prolongs stabilization of *N*-cadherin Blocks transition from the condensation state to the cartilaginous nodule formation
Wnt9a (14)	Expressed in developing joint interzone Expressed in synovium and joint capsule in the mature joint Arrests or reverses chondrogenic differentiation Blocks transition from the condensation state to the cartilaginous nodule formation
Fzl7	Inhibits mesenchymal condensation at the pre-cartilage aggregate formation by suppressing *N*-cadherin expression

**Table 2.** Role of Wnt signaling during late stages of cartilage development and adulthood.

**Promoting Effects**	
Wnt-4	Expressed in periphery of joint interzone and hypertrophic chondrocytes Accelerates chondrocyte terminal differentiation Induces terminal differentiation of growth plate cartilage
Wnt8	Promotes chondrocyte hypertrophy
β-catenin high level	Increases cell hypertrophy through RUNX-2 or IHH signaling activation Induces collagen X expression
**Inhibitory Effects**	
Wnt-5aWnt-5b	Inhibits hypertrophic maturation of chondrocytes through NF-κB stimulation and RUNX-2 inhibition
FRZB	Expressed in prechondrogenic mesenchymal condensations and in epiphyseal pre-articular chondrocytes Blocks chondrocyte maturation and prevents endochondral ossification *in vivo* Promotes glycosaminoglycan synthesis and expression of Sox9 and collagen II *in vitro*
DKK-1	Expressed at sites of programmed cell death in apical ectodermal ridge Expressed at higher level in articular cartilage than growth plate cartilage Promotes glycosaminoglycan synthesis and expression of Sox9 and collagen II *in vitro* Inhibits chondrocyte hypertrophy
Fzl1Fzl7	Blocks/delays chondrocyte hypertrophy
sFRP-1sFRP-3	Delays terminal differentiation of hypertrophic chondrocytes
